# Permeation characteristics of tetracyclines in parallel artificial membrane permeation assay

**DOI:** 10.5599/admet.657

**Published:** 2019-05-08

**Authors:** Sachika Yamauchi, Kiyohiko Sugano

**Affiliations:** Molecular Pharmaceutics Lab., College of Pharmaceutical Sciences, Ritsumeikan University, 1-1-1, Noji-higashi, Kusatsu, Shiga 525-8577, Japan

**Keywords:** Zwitterion, artificial membrane, permeability, phospholipid

## Abstract

The purpose of the present study was to characterize the passive permeation of tetracyclines in the parallel artificial membrane permeation assay (PAMPA). Tetracyclines exist as zwitterion at physiological pH. The PAMPA membrane was prepared by impregnating a phospholipid/decane solution to a filter support. The permeation coefficient (P_e_) of tetracycline (TC) was markedly affected by the lipid composition of the PAMPA membrane. No permeation was observed when phospholipid was not added (pure decane membrane, P_e_ < 0.05 × 10^-6^ cm/sec). With the addition of 2 % PC, little or no increase in P_e_ was observed. The addition of 1 % PE increased the P_e_ value more than tenfold. The addition of 2 % soybean lecithin containing phosphatidylinositol (PI) and phosphatidic acid (PA) increased the P_e_ value to above 4 × 10^-6^ cm/sec. The P_e_ value was further increased to 15 × 10^-6^ cm/sec by increasing the concentration of soybean lecithin from 2 to 10 %. The P_e_ value showed pH and temperature dependence, whereas it was not affected by the ionic strength, TC concentration, and ion-pair transport inhibitors. A weak correlation was observed between the P_e_ values and octanol-buffer distribution coefficients of tetracyclines. These results suggest that inter-molecular interactions between TC and PE, PI and/or PA facilitate the passive diffusion of TC across the PAMPA membrane.

## Introduction

Zwitterionic drugs are an important chemical class as they exhibit unique physicochemical and pharmacokinetic properties [1, 2]. A zwitterionic drug possesses at least one acidic group and at least one basic group (acidic p*K*_a_ < basic p*K*_a_). Typical examples of zwitterionic drugs are antibacterials, antiallergics, and diuretics. Zwitterion drugs are less liable to human ether-a-go-go related gene (hERG) and phospholipidosis compared to hydrophobic bases [3, 4]. According to the pH-partition theory, the passive lipid bilayer permeation of a zwitterionic drug is expected to be negligible. However, many zwitterionic drugs such as tetracyclines and fluoroquinolones show moderate to high passive permeability in vitro [5] and good bioavailability in vivo [6].

The parallel artificial membrane permeation assay (PAMPA) has been widely used as a high throughput assay for passive membrane permeation [7-10]. Interestingly, several zwitterionic drugs showed moderate to high permeability in PAMPA [6, 11]. In the case of cationic drugs, ion pair formation with an anionic phospholipid enhances the passive permeation of a drug [12,13]. However, the permeation mechanism of zwitterionic drugs has not been investigated. The purpose of the present study was to characterize the passive permeation of zwitterion drugs in PAMPA.

**Figure 1.**
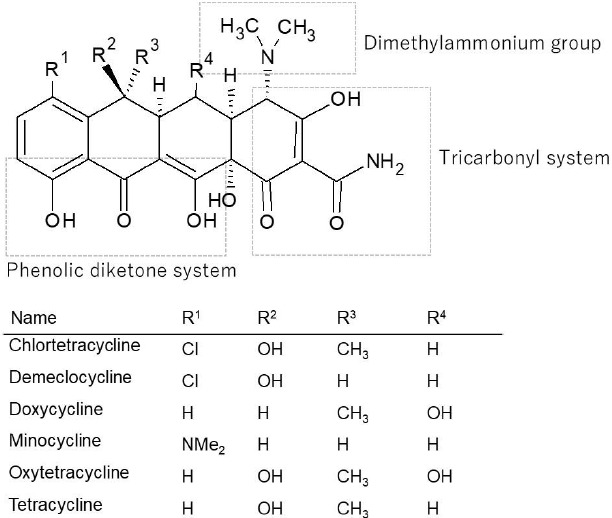
Chemical structures of tetracyclines

Tetracyclines were used as model zwitterionic drugs in this study. Tetracyclines have three dissociative functional groups (Figure 1) [14-16]. At the physiological pH in the small intestine, they predominantly exist as zwitterion [14]. The octanol buffer partition coefficients (log *D*) of tetracyclines are below 0.2 (Table 1) [17]. In addition, they violate the Lipinski’s rule of five in the hydrogen bond number [18]. However, tetracyclines show good oral absorption in vivo [19]. Interestingly, tetracycline showed moderate permeability in the biomimetic PAMPA [9].

## Experimental

### Materials

Tetracycline hydrochloride, L-leucine, decane, sodium dihydrogen phosphate, sodium chloride, phosphatidylethanolamine (PE), and 8N NaOH were purchased from Wako Pure Chemical Industries, Ltd (Osaka, Japan). Oxytetracycline hydrochloride, minocycline hydrochloride, doxycycline hyclate, and 2-aminooctanoic acid were purchased from TCI (Tokyo, Japan). Demeclocycline hydrochloride and chlortetracycline hydrochloride were purchased from LKT Labs, Inc (MN, USA). Phosphatidylcholine (PC) was purchased from NOF corporation (Tokyo, Japan). Tetrahexylamine bromide (THA) was purchased from Sigma-Aldrich Co. LLC (MO, USA). Procainamide hydrochloride was purchased from Combi-Blocks Inc (CA, USA). Soy bean lecithins (SLP-PC 70, SLP-white, SLP-PI grades) were provided by Tsuji Oil Mills co., Ltd (Mie, Japan).

### Methods

#### PAMPA assay

The PAMPA sandwich was consisted of a 96 well filter plate (hydrophobic PVDF, 0.45 μm) and a PAMPA acceptor plate (Merck Millipore, MA, USA). Before forming the PAMPA sandwich, the bottom (acceptor) plate was filled with 300 μL of a 50 mM sodium phosphate buffer. The filter of the top (donor) compartment was coated with 5 μL of a phospholipid – decane solution. The compositions of soy bean lecithins were shown in Table 2. The buffer conditions were the same for both donor and acceptor compartments (iso-pH and iso-ionic strength condition). The PAMPA sandwich was placed in a plastic container containing a small amount of water on the bottom and incubated for 3 h (at 25 and 37 °C) or 18 h (at 15 °C). After incubation, 100 μL of both the donor and acceptor solutions were transferred to UV plates. The concentrations of tetracyclines and procainamide were measured at 360 and 280 nm, respectively. The PAMPA permeability was calculated by the following equation [19]:


(1)

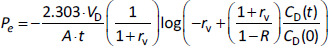




(2)

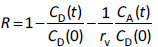




(3)

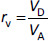



where *P_e_* is the effective permeation coefficient (cm/s), *A* is the filter surface area (0.266 cm^2^), *V*_D_ and *V*_A_ are the volumes in the donor and acceptor phase (0.15 and 0.3 mL, respectively), *t* is the incubation time, *C*_D_(t) is the concentration of a drug in the donor phase at time *t*, *R* is the membrane retention factor, and *r*_v_ is the volume ratio.

#### Octanol-buffer distribution coefficient

The octanol-buffer distribution coefficients (log *D*) of tetracyclines were determined by a shake-flask method at pH 6.5 (50 mM sodium – phosphate buffer). The octanol and buffer phases were mutually pre-saturated before use. A buffer solution of a model drug (1.0 mM, 0.5 mL) and octanol (2.5 mL) were added to a 15 mL tube. The sample was vigorously shaken for 60 min at room temperature. The concentrations of tetracyclines were determined by UV spectroscopy at 360 nm.

## Results

### Effect of membrane composition

**Figure 2.**
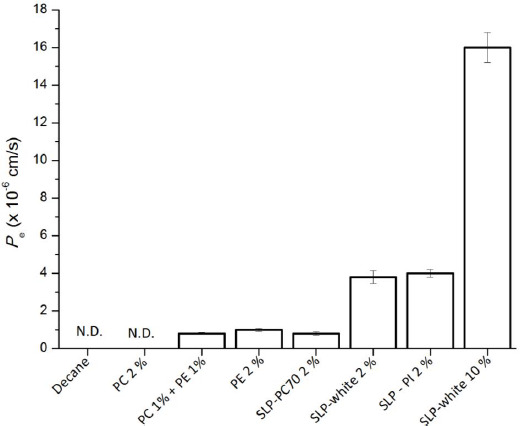
Effect of membrane composition on PAMPA permeation of tetracycline (mean ± SD, n = 3-9). Assay conditions: TC 0.5 mM, 10 % SLP-white/decane, pH 6.5 50 mM sodium phosphate buffer, 37 °C.

The PAMPA permeation of tetracycline (TC) was markedly affected by the lipid composition of the PAMPA membrane (Figure 2). No permeation was observed when phospholipid was not added (pure decane membrane, *P*_e_ < 0.05 × 10^-6^ cm/sec). With the addition of 2 % PC, little or no increase in *P*_e_ was observed. The addition of 1 % PE increased the *P*_e_ value more than tenfold. The addition of 2 % soybean lecithin containing phosphatidylinositol (PI) and phosphatidic acid (PA) increased the *P*_e_ value to above 4 × 10^-6^ cm/sec. The *P*_e_ value was further increased to 15 × 10^-6^ cm/sec by increasing the concentration of soybean lecithin from 2 to 10 %. The 10% soybean lecithin (SLP white) - decane membrane was used in the following studies.

### pH and ionic strength dependency

**Figure 3.**
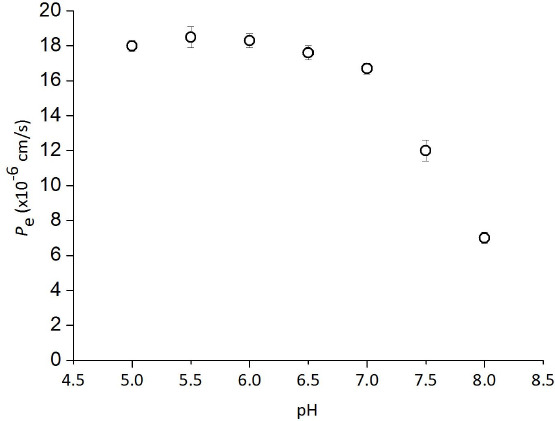
Effect of pH on PAMPA permeation of tetracycline (mean ± SD, n = 3). Assay conditions: TC 0.5 mM, 10 % SPL-white / decane, 50 mM sodium phosphate buffer, at 37 °C.

**Figure 4.**
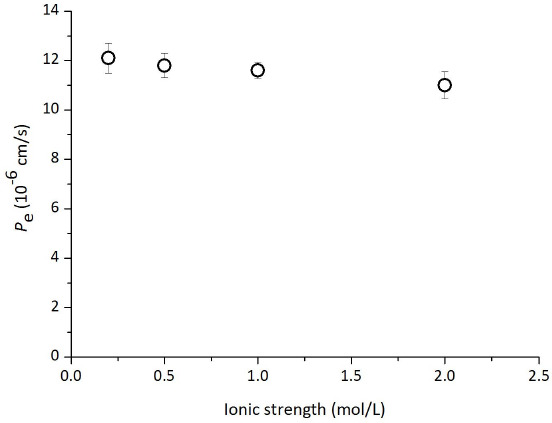
Effect of ionic strength on PAMPA permeation of tetracycline (mean ± SD, n = 3). Assay conditions: TC 0.5 mM, 10 % SPL-white / decane, pH 6.5, 37 °C. The ionic strength of the medium was adjusted by NaCl.

The effect of pH on the PAMPA permeation of TC is shown in Figure 3. The *P*_e_ values decreased as pH was increased above 7.0. The effect of the ionic strength (*I*) is shown in Figure 4. The ionic strength showed little or no effect in the range of *I* = 0.15 to 2.0 M.

### Temperature dependence

**Figure 5.**
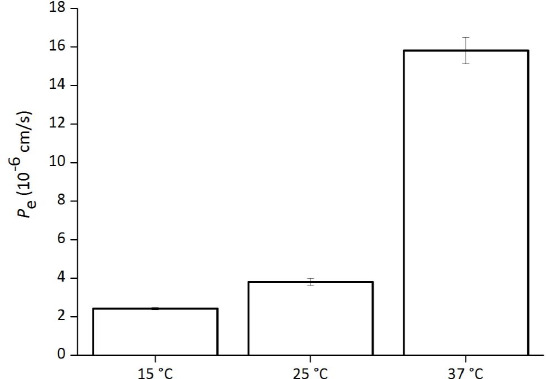
Effect of temperature on PAMPA permeation of tetracycline (mean ± SD, n = 3). Assay conditions: TC 0.5 mM, 10 % SPL-white / decane, pH 6.5 50 mM sodium phosphate buffer.

The effect of temperature on the PAMPA permeation of TC is shown in Figure 5. As the temperature was increased from 15 °C to 37 °C, the *P*_e_ value was increased 6.5 fold.

### Concentration dependence

**Figure 6.**
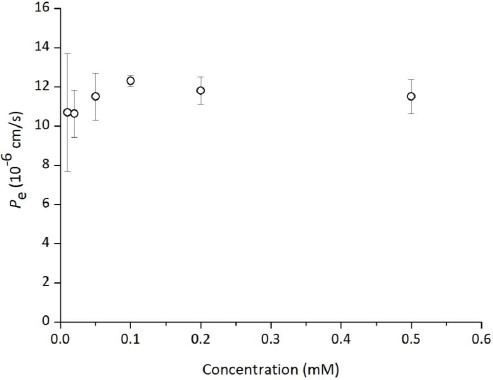
Effect of tetracycline concentration on PAMPA permeation (mean ± SD, n = 3). Assay conditions: 10 % SPL-white / decane, pH 6.5 50 mM sodium phosphate buffer, 37 °C.

The effect of TC concentration on the PAMPA permeation is shown in Figure 6. The *P*_e_ value of TC was not affected by the TC concentration up to 0.5 mM. Due to the solubility of TC in the medium, the concentration of TC could not be increased above 0.5 mM.

### Effect of additives in the donor media

**Figure 7.**
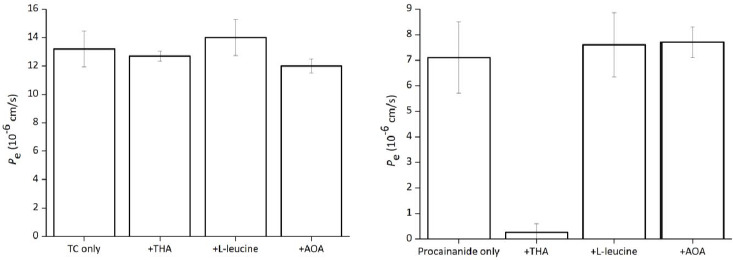
Effect of additives on PAMPA permeation of tetracycline and procainamide (mean ± SD, n = 3). Assay conditions: substrates 0.5 mM, additives 10 mM, 10 % SPL-white / decane, pH 6.5 50 mM sodium phosphate buffer, 37 °C.

The effect of possible inhibitors on the PAMPA permeation of TC is shown in Figure 7. Procainamide was used as a control of the ion-pair transport (a cation drug and an anion phospholipid). The permeation of procainamide was inhibited by tetrahexylammonium (THA), however not by L-leucine and 2-amino octanoic acid (AOA). The permeation of TC was not inhibited by the inhibitors employed in this study.

### Log P_e_ – log D relationship

**Figure 8.**
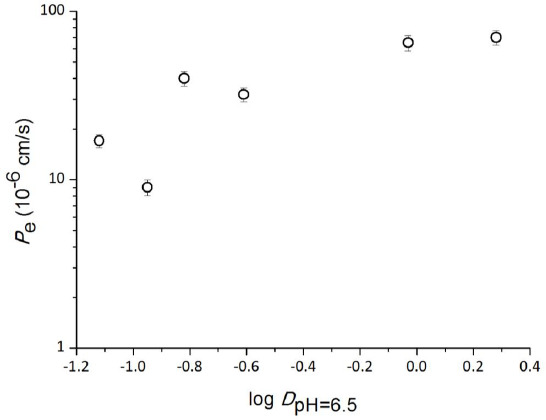
Correlation between log *D* and PAMPA permeability for tetracyclines (mean ± SD, n = 6). PAMPA conditions: tetracyclines 0.5 mM, 10 % SPL-white / decane, pH 6.5 50 mM sodium phosphate buffer, 37 °C.

The log *P*_e_ – log *D* relationship for tetracyclines is shown in Table 3 and Figure 8. A weak correlation was observed between log *P*_e_ and log *D*.

## Discussion

We first investigated the effect of the membrane composition on the PAMPA permeation of TC. The results of the lipid composition dependency study suggest that intermolecular interactions between TC and PE, PS and/or PI facilitate the passive diffusion of TC across the PAMPA membrane [20]. The results of this study are in good agreement with the previous finding that the lipid composition is critically important for the PAMPA assay [9,21]. The *P_e_* value of TC in the 10 % soybean lecithin/decane membrane was similar to that observed in the biomimetic PAMPA in which 1,7-octadiene was used as an organic solvent. Even though the biomimetic PAMPA showed promising predictability for in vivo oral drug absorption, as 1,7-octadiene is irritant, it is not suitable for routing use.

TC predominantly exists as zwitterion form (net zero charge) in the range of pH 4.0 to 7.0. In this pH range, the *P_e_* value remained constant. Above pH 7.0, the *P*_e_ value decreased as TC becomes negatively charged (two anions and one cation) due to the dissociation of the phenolic diketone part above pH 7.0 (Figure 1) [22]. However, the inflection pH point was below the p*K*_a_ of TC. In addition, the slope of the pH - log *P_e_* line above pH 7.0 was about -0.5. These deviate from the theoretical pH – log *P*_e_ curve based on the pH partition theory. Further investigation is required to clarify the reasons for these deviations. Ionic strength had no effect on the *P*_e_ value, suggesting that TC did not form an ion pair with the inorganic ions in the buffer at pH 6.5.

The PAMPA permeation of TC showed marked temperature dependence in this study. The ratio of *P*_e_ values between 15 °C and 37 °C was 6.5. This ratio was greater than previously reported by Vizserálek et al. for undissociable, mono-acid, and mono-base drugs using the 2 % PC/1 % cholesterol/dodecane membrane (1.1 to 3.7) [23].

To investigate the permeation mechanism of TC, the saturation and inhibition of TC permeation were investigated. Neither saturation nor inhibition was observed. This result was different from the results for hydrophilic basic drugs, for which both saturation and inhibition have been reported [12]. For hydrophilic basic drugs, ion pair formation between a drug cation and an anionic phospholipid was suggested as the permeation mechanism [12, 13]. Since the addition of PE, PI and/or PA significantly enhanced the *P*_e_ value of TC, there might exist some nonspecific inter-molecular interaction between TC and these phospholipids other than ion-pair formation [20]. Only a weak correlation was observed between log *D* and log *P*_e_, suggesting that the inter-molecular interaction between phospholipids and tetracyclines might be different from that between octanol and tetracyclines.

## Conclusions

In conclusion, in this study, the permeation characteristics of TC were investigated in detail. The phospholipid composition and incubation temperature showed marked effects on the permeation of TC, whereas the ionic strength of the media, the concentration of TC, and the addition of ion pair inhibitors showed little or no effect.

## Figures and Tables

**Table 1.** Physicochemical properties of tetracyclines

	MW	p*K*_a_	log *D* (pH 6.5)^a^	p*K*_a_ Ref.
Chlortetracycline	479	3.3, 7.6, 9.3	-0.88	(14) ^b^
		3.25, 6.72, 8.84		(16) ^c^
Demeclocycline	465	3.4, 7.4, 9.4	-0.67	(14) ^b^
Doxycycline	444	3.0, 8.0, 9.2	-0.08	(14) ^b^
		3.50, 7.25, 9.58		(16) ^c^
Minocycline	457	2.8, 5.0, 7.8, 9.5	0.20	(15) ^d^
Oxytetracycline	460	3.2, 7.5, 8.9	-0.96	(14) ^b^
		3.53, 7.25, 9.58		(16) ^c^
Tetracycline	444	3.3, 7.8, 9.6	-1.09	(14) ^b^
		3.35, 7.29, 9.88		(16) ^c^

^a^ Measured in this study.

^b^ Potentiometry (23 °C), ionic strength = 0.01 or 0.05 M.

^c^ Potentiometry (25 °C), ionic strength = 0.1 M.

^d^ Method not described in the literature.

**Table 2.** Lipid composition of soy bean lecithin^a^

Phospholipid	SLP-PC70 (%)	SLP-white (%)	SLP-PI (%)
Phosphatidylcholine (PC)	65 – 75	24 – 32	15 – 22
Phosphatidylethanolamine (PE)	10 – 15	20 – 28	25 – 32
Phosphatidylinositol (PI)	0 – 1	12 – 20	18 – 25
Phosphatidic acid (PA)	1 – 3	8 – 15	8 – 15
Lysophosphatidylcholines (LPC)	1 – 5	1 – 5	1 – 5

^a^ Taken from the product information provided by the manufacturer.

**Table 3.** PAMPA permeability for tetracyclines^a^

	*P*_e_ (10^-6^ cm/sec, mean ± SD, n = 6)
Chlortetracycline	41 ± 3
Demeclocycline	33 ± 1
Doxycycline	64 ± 5
Minocycline	71 ± 6
Oxytetracycline	9.2 ± 0.7
Tetracycline	15 ± 1

^a^ Tetracyclines 0.5 mM, 10% SPL-white / decane, pH 6.5 50 mM sodium phosphate buffer, 37 °C.
